# Maintenance of Cell Wall Integrity under High Salinity

**DOI:** 10.3390/ijms22063260

**Published:** 2021-03-23

**Authors:** Jianwei Liu, Wei Zhang, Shujie Long, Chunzhao Zhao

**Affiliations:** 1 Shanghai Center for Plant Stress Biology, CAS Center for Excellence in Molecular Plant Sciences, Chinese Academy of Sciences, Shanghai 200032, China; jwliu@psc.ac.cn (J.L.); weizhang@psc.ac.cn (W.Z.); shjlong@psc.ac.cn (S.L.); 2 University of the Chinese Academy of Sciences, Beijing 100049, China

**Keywords:** cell wall integrity, cell wall sensor, salt stress, salt tolerance, LRXs, *Cr*RLK1Ls

## Abstract

Cell wall biosynthesis is a complex biological process in plants. In the rapidly growing cells or in the plants that encounter a variety of environmental stresses, the compositions and the structure of cell wall can be dynamically changed. To constantly monitor cell wall status, plants have evolved cell wall integrity (CWI) maintenance system, which allows rapid cell growth and improved adaptation of plants to adverse environmental conditions without the perturbation of cell wall organization. Salt stress is one of the abiotic stresses that can severely disrupt CWI, and studies have shown that the ability of plants to sense and maintain CWI is important for salt tolerance. In this review, we highlight the roles of CWI in salt tolerance and the mechanisms underlying the maintenance of CWI under salt stress. The unsolved questions regarding the association between the CWI and salt tolerance are discussed.

## 1. Introduction

High salinity is an adverse environmental stress that severely affects the growth and yield of crops. Excessive accumulation of sodium in plants confers both ion toxicity and osmotic stress, which in turn dramatically affect the morphological, physiological, biochemical, and metabolic status of plants [1]. Currently, more than 20% of the irrigated lands in the world are threatened by high salinity, and the area of saline soils is increasing gradually every year accompanied by the global climate change and poor irrigation practices [2,3,4]. It is expected the global population will reach to nearly 10 billion in 2050, and to meet the increasing food demand in future, the utilization of saline soils to grow major crops tends to be inevitable. Therefore, the cultivation of crops with increased salt tolerance is a major objective in salt stress community.

To avoid the damage caused by excessive salts in soil, plants have evolved various strategies to overcome the problems caused by high salinity. Ion homeostasis, osmotic adjustment, ROS balance, and metabolic adjustment are the major factors that are associated with the tolerance of plants to salt stress. Based on the capacity of plants to adapt to salt stress, plants can be classified into glycophytes and halophytes. Our major crops, such as rice, maize, and wheat, are glycophytes that are unable to complete their life cycle when they are being exposed to high salinity. Halophytes, however, have developed various strategies to adapt to the environments with a high concentration of sodium. For example, halophytes are able to extrude salts via glands or store excessive Na^+^ in the vacuoles of epidermal bladder cells [5,6].

More and more studies point out that maintenance CWI is also critical for the adaptation of plants to high salinity. Plant cell walls, which mainly consist of polysaccharides and structural proteins, are essential for the establishment of plant morphology and protection of plants against adverse environmental changes [7]. During plant growth and development or in response to environmental stresses, the cell wall compositions and structures are dynamically modulated, allowing rapid cell elongation and increased stress tolerance [8]. To maintain CWI during the reorganization of cell wall, plants need to constantly monitor the chemical and mechanical properties of the cell walls and also need to process an ability to repair cell wall once they are seriously disrupted. It has been shown that CWI maintenance mechanism exists in plants and is essential for the regulation of growth and development and in response to stress conditions [9,10]. The progresses about CWI sensing and maintenance system in plants have been summarized in several outstanding review papers [8,11,12]. In this review, we focus on the elucidation of the associations between CWI and salt tolerance in plants.

## 2. Importance of Cell Wall Biosynthesis in Salt Tolerance

The plant cell wall is a dynamic network composed of cellulose, hemicellulose, pectin, lignin, and multiple types of structural proteins [13,14]. Moreover, cell wall-remodeling enzymes, various ions, and reactive oxygen species (ROS) also exist in the apoplast and are involved in the regulation of CWI. Upon exposure to high salinity, several changes in the cell wall have been identified, including the reduction of cellulose content [15,16], disruption of the cross-linking of pectins [9], and accumulation of lignin [17]. Studies have shown that the plants that are defective in cell wall biosynthesis are hypersensitive to salt stress, suggesting that maintenance of CWI is important for the adaptation of plants to high salinity.

### 2.1. Cellulose

Cellulose is the most abundant organic component in the cell wall of terrestrial vascular plants. Cellulose micro-fibrils are composed of β-1,4-linked glucan chains, which are synthesized at the cell surface by cellulose synthase (CesA) complexes (CSCs) [18,19]. Each CSC is assembled into a hexameric rosette structure, harboring CesA catalytic subunits and several accessory proteins. In *Arabidopsis*, there are ten CesA proteins [18]. It is well known that CesA1, CesA3, and CesA6 are assembled in a CSC to synthesize cellulose in the primary cell wall, while CesA4, CesA7, and CesA8 are mainly involved in the synthesis of cellulose in the secondary cell wall [20]. Experimental data have shown that the cellulose contents are significantly reduced after salt treatment and the plants with a loss of function of CESA1 and CESA6 gene display reduced root elongation and severe root tip swelling under salt stress, indicating that cellulose biosynthesis is important for salt tolerance in plants [21,22]. Clear evidences have indicated that the CSCs are dissociated from plasma membrane within 30 min after exposure to high salinity. However, during the growth recovery phase after salt treatment, the CSCs can be reassembled at the plasma membrane to synthesize new cellulose, and the capacity to reassemble CSCs during the growth recovery stage is critical for plants to maintain root and hypocotyl growth under salt stress [16].

Apart from the CesAs, several cellulose biosynthesis-related proteins have also been reported involved in salt tolerance. For example, KORRIGAN1 (KOR1), a putative endo-1,4-β-D-glucanase, is an integral part of the primary cell wall CSC and is required for root elongation under salt stress [22,23]. Cellulose synthase interacting protein 1 (CSI1) and companion of cellulose synthase 1 (CC1 and CC2) proteins, acting as companions of CesAs, are both required for cellulose biosynthesis [16,21]. Mutations in *CSI1* or *CC1* and *CC2* lead to reduced root or hypocotyl elongation under salt stress. *CTL1* encodes a chitinase-like protein that participates in the deposition of the ordered cellulose, and mutation of this gene results in increased sensitivity to high salinity [24] (Table 1).

### 2.2. Hemicellulose

Hemicelluloses are grouped into xyloglucans (XyG), xylans, mannans, and β-(1,3;1,4)-glucans, and the abundance and structure of these polysaccharides vary greatly in different plants species [35]. Xylan is considered as a cross-linking polysaccharide in the establishment of cell wall architecture [35,36]. XyG contributes to the strengthening of cell wall during cell elongation by binding to cellulose micro-fibrils with hydrogen bonds [37,38]. XyG can be cleaved by the cell wall remodeling enzymes xyloglucan endotransglucosylase/hydrolases (XTHs) [39]. After cleavage, the reducing end of the XyG is attached to the non-reducing end of another XyG oligomer or polymer to produce chimeric XyG molecules [39]. The XTHs-mediated modification of XyG is considered to be important for controlling cell wall extensibility. Studies have reported that XTHs are involved in salt stress response in plants. *Arabidopsis XTH30*, encoding a xyloglucan endotransglucosylase/hydrolase 30, is strongly upregulated under salt stress [30]. Loss of function of the *XTH30* gene leads to increased salt tolerance, which is mainly caused by the slower reduction of crystalline cellulose content and alleviated depolymerization of microtubules in response to salt stress [30]. This result suggests that XTH30 plays a negative role in salt tolerance. However, the positive roles of XTHs in salt tolerance have also been reported. Constitutive expression of *CaXTH3* in hot pepper [40,41] and *PeXTH* in *Populus euphratica* [42] enhance tolerance to salt stress, and disruption of *XTH19* and *XHT23* genes in *Arabidopsis* results in decreased salt tolerance [43].

### 2.3. Pectin

Pectin is a group of acidic polysaccharides that are enriched with α-(1, 4)-linked galacturonic acids in the backbone [44]. Pectin accounts for up to 40% of the dry weight of higher plant cell walls [44] and plays critical roles in plant growth and development [45], leaf senescence [46], biotic [47] and abiotic stress responses [48]. Pectin is composed of three major types: homogalacturonan (HG), rhamnogalacturonan-I (RG-I), and rhamnogalacturonan-II (RG-II) [7,44]. HG is synthesized in the Golgi apparatus and secreted to the apoplast in a highly methy-esterified form and later it is selectively de-esterified by pectin methyl esterases (PMEs) during cell growth and in response to environmental stimuli [7,44]. The degree and pattern of the methyl-esterification of pectin in some extent determines the stiffness of cell walls [49]. In *Arabidopsis*, there are around 66 members of PME family protein, and for most of PMEs, their activities can be inhibited by endogenous PME inhibitors (PMEIs) or a natural inhibitor epigallocatechin gallate (EGCG) [50,51]. High salinity triggers the demethyl-esterification of loosely bound pectins to inhibit cell swelling [52] and previous studies showed that the activity of PMEs is either positively or negatively associated with salt tolerance in plants [53]. For instance, null *Arabidopsis* function mutant *pme13* is hypersensitive to Na^+^ toxicity in seed germination and seedling growth [53]. In contrast, overexpression of *Chorispora bungeana PMEI1* or *AtPMEI13* in *Arabidopsis* causes decreased PMEs activity and enhanced methyl-esterification level of pectins, which subsequently improves seeds germination and survival rate under salt stress [31]. The de-esterified HG molecules can be cross-linked to form the so called egg-box structure, the process of which is mediated by divalent cations, such as Ca^2+^, and the formation of egg-box structure promotes cell wall stiffening [54]. In the presence of high concentration of Na^+^, the ratio of Na^+^/Ca^2+^ in the apoplast is increased, and Na^+^ is supposed to replace Ca^2+^ to bind pectins and thus disturbs the cross-linking of pectins, leading to reduced cell elongation [55]. Besides, the borate-mediated cross-linking of RG-II contributes to the strength of cell wall and is required for the regulation of growth recovery after exposure to high salinity [56,57].

The roles of pectin in salt tolerance have also been reported in rice. Polygalacturonase 1 (PG1) is a cell wall hydrolase that is responsible for the degradation of cell wall pectin. Overexpression of *OsBURP16*, which encodes a non-catalytic β subunit of PG1, results in an increased pectin degradation and increased salt-hypersensitivity in rice [34]. *OsTSD2* encodes a pectin methyltransferase in rice, and mutation in *OsTSD2* leads to a higher content of Na^+^ and a lower level of K^+^ in rice shoot under high salinity, which is mainly caused by the reduced expression of genes that are responsible for the maintenance of ion homeostasis, such as *OsHKT1;5*, *OsSOS1*, and *OsKAT1* [33] (Table 1).

### 2.4. Lignin

As one of the most abundant organic compound in plants, lignin is composed of phenylalanine-derived [58] or tyrosine-derived [59] aromatic monomer substances and is important for the secondary cell wall formation and the responses to a variety of environmental stresses [60]. High salinity induces the accumulation of lignin content and cell wall thickening via the activation of lignin biosynthesis pathway [60]. The accumulation of lignin contributes to the mechanical strengthening of cell wall and protection of membrane integrity under salt stress [61]. The effects of lignin accumulation on salt tolerance have been reported in different crops, including soybean [62], wheat [63], and tomato [64]. *CCoAOMT* encodes a caffeoyl CoA *O*-methyltransferase (CCoAOMT), which catalyzes caffeoyl CoA to feruloyl CoA in lignin biosynthesis pathway. The expression of *CCoAOMT* is induced in salt-adapted cell, and the plants with a loss-of-function of *CCoAOMT* are hypersensitive to salt stress [17]. *BpMYB46* and *BpNAC012*, encoding two transcription factors in white birch (*Betula platyphylla*), are required for the up-regulation of lignin biosynthetic genes and salt stress-responsive genes, and overexpression of these two genes enhances salt tolerance in *B. platyphylla* [65,66]. AgNAC1, a nuclear-localized protein in celery, acts as a positive regulator in inducing the expression of lignin-related and salt stress-responsive genes, and overexpression of *AgNAC1* enhances the formation of secondary walls and plant salt tolerance [67].

## 3. The Roles of the Cell Wall-Localized Glycoproteins in Salt Stress Response

In addition to dynamic and complex polysaccharide networks, several types of cell wall proteins (CWPs) have been identified in the apoplast. CWPs play critical roles in cell wall modifications and cell wall stress signals transduction. Hydroxyproline (Hyp)-rich glycoproteins (HRGPs), proline-rich proteins (PRPs), glycine-rich proteins (GRPs), and arabinogalactan proteins AGPs are the major types of CWPs [68]. For most of CWPs, they are secreted into the apoplast in a glycosylation-modified form [69,70,71].

Extensins (EXTs) are a group of cell wall glycoproteins that belong to the HRGPs family. EXTs are typically characterized for the enrichment of Ser-(Hyp)_3–5_ repeats in their protein sequences [72], and each Hyp residue is decorated with up to five arabinose units by several different arabinosyltransferases, including HPAT1-HPAT3 [73], RRA1-RRA3 [74], XEG113 [75], and ExAD [76]. The arabinosylation of EXTs is suggested to be important for the fulfillment of their biological functions. Our recent study showed that the mutation of *MUR4*, which encodes an UDP-Xyl 4-epimerase that is essential for the conversion of UDP-Xyl to UDP-Ara*p* in Golgi, results in reduced root elongation under salt stress, suggesting that arabinose biosynthesis and subsequently the modification of polysaccharides and glycoproteins by arabinose are important for salt tolerance in plants [27].

**Figure 1:**
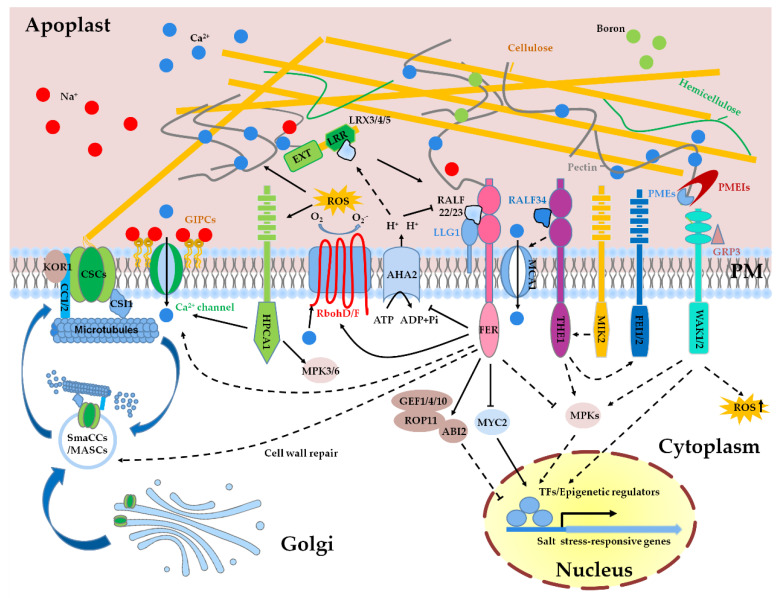
Sensing and maintenance of cell wall integrity under salt stress. Salt stress-induced cell wall changes are proposed to be sensed by multiple receptor-like kinases, including FER, THE1, MIK2, FEI1/2, and WAK1/2. As one of the most important cell wall integrity (CWI) sensors, FER may function alone or together with LRX3/4/5-RALF22/23 module to perceive the perturbation of CWI caused by high salinity. The AHA2-mediated acidification of the apoplastic pH increases the affinity of LRXs with RALFs, while the alkaline state in the apoplast promotes the binding of RALFs with FER. FER and probably also other cell wall sensors convert salt-triggered cell wall signals to multiple intracellular signals, including Ca^2+^, ROS, abscisic acid (ABA), jasmonic acid (JA), and MPKs, which in turn regulate the expression of salt stress-responsive genes in the nucleus. Salt stress can alter the redox status in the apoplast, and RbohD/F-mediated production of the apoplastic H_2_O_2_ may affect the cross-linking of cell wall polymers and activate H_2_O_2_ sensor HPCA1. Glycosyl inositol phosphorylceramide (GIPC) sphingolipids participate in the sensing of extracellular salt by directly binding to sodium ions. Cell wall biosynthesis- and modification-related components, including pectin methyl esterases (PMEs), PME inhibitors (PMEIs), and cellulose synthase (CesA), are involved in the regulation of salt tolerance in plants. Upon initial exposure to salt stress, cortical microtubules are depolymerized and cellulose synthase complex (CSC) together with its companions CSI1 and CC1/2 are internalized into small CesA compartments/microtubule-associated CesA compartments (smaCCs/MASCs). At the growth recovery stage after salt application, FER is probably required for the regulation of the reassembly of cortical microtubules and the relocation of CSCs to the plasma membrane to synthesize cellulose, which subsequently enhances the adaptation of plants to salt stress. Solid lines represent direct regulations, and dashed lines represent in-direct or potential regulations.

Leucine-rich repeat extensins (LRXs) are chimeric proteins that contain an N-terminal leucine-rich repeat (LRR) domain that binds with interacting partners and a C-terminal extensin domain that is likely linked with the EXT network or polysaccharides in the apoplast [77]. *LRXs* gene family consists of 11 members in *Arabidopsis*, among of which *LRX3*, *LRX4*, and *LRX5* are dominantly expressed in vegetative tissues [77]. The biological functions of these three LRX proteins are redundant, as mutation of each single gene does not cause any obvious phenotypes, but *lrx34* double and *lrx345* triple mutants both exhibit dwarfism, increased accumulation of anthocyanin, and increased sensitivity to high salinity [10]. It is worth noting that all these phenotypes are more severe in the *lrx345* triple mutant than that in the *lrx34* double mutant. Our study indicated that *fer-4* mutant as well as the transgenic plants overexpressing *RALF22* and *RALF23* exhibit similar phenotypes as *lrx345* in terms of plant growth and salt sensitivity, and biochemical data show that RALF22 and RALF23 are physically associated with LRX3/4/5 [10]. Combining the data showing that FER is the receptor of RALFs [9], we can conclude that the LRX3/4/5, the secreted peptide RALFs, and the receptor-like kinase FER function as a module to mediate salt stress response in the apoplast. It is supposed that the extensin domain of LRXs is able to anchor polysaccharides in the cell wall [77,78], but it is still unknown whether LRXs directly participate in the sensing of CWI or coordinate with FER to perceive CWI (Figure 1).

AGPs are highly glycosylated with arabinogalactan chains and are proposed to play important roles in salt stress response [69]. Our study showed that the reduced root elongation of the *mur4* mutant under high salinity is partially caused by the decreased AGPs, as application of gum arabic, a commercial source of *Acacia senegal* AGPs, restores the root elongation of the *mur4* mutant under salt stress [27]. As a glycosylphosphatidylinositol (GPI)-anchored fasciclin-like AGP, salt overly sensitive 5 (SOS5)/fasciclin-like arabinogalactan-protein 4 (FLA4) was identified based on a screening of mutants with increased sensitivity to salt stress. The *sos5/fla4* mutant exhibits reduced root elongation and severe root tip swelling under salt stress [79,80]. SOS5 is glycosylated by galactosyltransferase 2 (GALT2) and GALT5, both of which belong to AGP-specific galactosyltransferases. The *galt2 galt5* double mutant displays a similar phenotype as the *sos5/fla4* mutant in the presence of high concentration of NaCl [80]. Recently, studies showed that AGPs are able to cross-link with cell wall components. For instance, arabinoxylan pectin arabinogalactan protein 1 (APAP1) is covalently linked to pectins [81], and arabinogalactan protein 31 (AGP31) physically associates with methyl-esterified polygalacturonic acid and galactans, which are the branches of RG-I [82].

Expansins, first isolated from growing cucumber hypocotyls, consist of four subfamilies: α-expansin, β-expansin, expansin-like A, and expansin-like B [83,84]. Expansins are key regulators of cell-wall loosening and are required for cell enlargement under a variety of environmental stresses [85]. Several studies have shown that the expression of expansin-encoding genes is induced by high salt and the elevation of the protein levels of expansins tends to promote salt tolerance in plants. *ZmEXPB2*, *ZmEXPB6*, and *ZmEXPB8* genes in maize [86], *AsEXP1* gene in turf grass [87], and *OsEXPA3* gene in rice [88], are induced upon exposure to high salinity. Down-regulation of *ZmEXPB6* is correlated with the reduced leaf growth of maize under salt stress [89]. Overexpression of rice *expansin 7* (*OsEXPA7*) confers substantially enhanced tolerance to salt stress by lowering reactive oxygen species (ROS) accumulation and increasing antioxidant activity in rice [90]. Ectopic expression of wheat *expansin 2* (*TaEXPA2*) or *TaEXPB23* improves salt tolerance in tobacco [91,92]. Although expansins have been known to positively regulate salt stress response in multiple species, few studies have revealed the mechanisms underlying the expansins-mediated regulation of salt tolerance.

## 4. Salt Stress Alters the Redox Status in the Apoplast

Reactive oxygen species (ROS) are a class of metabolites, including hydrogen peroxide, singlet oxygen, superoxide, and hydroxyl radicals, which are produced in chloroplasts, mitochondria, peroxisomes, and apoplast [93]. The salt stress-triggered production of ROS and their effects on CWI have been widely reported in plants [93,94,95]. ROS triggers the cross-linking of cell wall compounds and enhances the mechanical strength of cell wall under a short-term stress exposure. Under a prolonged stress treatment, the formation of hydroxyl radicals (•OH) cleave plant polysaccharides, leading to cell wall loosening [96]. The ROS-induced lignin biosynthesis facilitates the adaptation of plants to high salt environment [95,97].

The production of ROS in the apoplast is mainly mediated by respiratory burst oxidase homolog D (RbohD) and RbohF [98], two NADPH oxidases that are localized at the plasma membrane. NADPH oxidases transfer electrons from cytosolic NADPH or NADH to apoplastic oxygen, leading to the production of superoxide (O_2_^−^), which is then catalyzed to hydrogen peroxide (H_2_O_2_) by superoxide dismutases [99]. The expression of *RbohD* and *RbohF* is induced under salt stress and *rbohD rbohF* double mutant is hypersensitive to salt stress [100], suggesting that the ROS production in the apoplast is required for salt tolerance. Salt-induced production of ROS by RbohD/F is able to activate Ca^2+^ channel to increase the influx of Ca^2+^ into cytosol, which mediates the modulation of Na^+^/K^+^ homeostasis [100]. The H_2_O_2_ generated by RbohD/F during the early stage of stress treatment also acts as a signal molecule to activate antioxidant system to attenuate salt stress-induced oxidative damages [101]. Recent studies showed that RbohD/F form nanoclusters at the plasma membrane in response to osmotic stress and later they are internalized into the cytoplasm via membrane microdomains [102,103,104]. As high salt conditions are accompanied by osmotic stress, the formation of RbohD/F as nanoclusters at the plasma membrane is perhaps also the case in the plants being exposed to high salinity (Figure 1).

Class III peroxidases are heme-containing enzymes, which are mainly localized in the apoplast and vacuole. Class III peroxidases either positively or negatively modulate apoplastic ROS levels [105]. Class III peroxidases explore H_2_O_2_ and O_2_^−^ to generate •OH, which leads to the cleavage of polysaccharides and promotes cell wall loosening [106]. Class III *peroxidase 71* (*PRX71*), which is strongly up-regulated in response to cell wall damage (CWD), negatively regulates growth and cell size and positively regulates ROS accumulation [94]. *GsPRX9*, encoding a Class III peroxidase, is induced by salt treatment in soybean root, and the soybean transgenic plants overexpressing *GsPRX9* exhibit increased root growth and decreased H_2_O_2_ content under salt stress [107].

The biological significance of the salt stress-induced redox change in the apoplast is still far from being fully understood. One of the outputs of the redox change is to affect the formation of intra- and inter-molecular disulfide bond. A large number of cell wall-localized glycoproteins and secreted peptides are characterized with the enrichment of cysteines, which are potentially involved in the formation of disulfide bonds. Therefore, we can speculate that the salt stress-induced redox change can affect the intra- and inter-molecular disulfide bridges of cell wall glycoproteins, which in turn transduce cell wall signals to the cellular interior. LRX8 and RALF4, which are both required for the regulation of pollen tube growth, process cysteines that are involved in the formation of disulfide bridges. A recent structural study showed that the formation of LRX8 homodimer and also the physical association of RALF4 with LRX8 require oxidative environment. Abolishment of the disulfide bonds via sites mutation or treatment of proteins with dithiothreitol (DTT) largely prevents the formation of LRX8 homodimer and affects the affinity of LRX8 with RALF4 [108]. These results suggest that the redox status in the cell wall is required for the regulation of the formation of LRXs-RALFs complex. Based on this hypothesis, we propose that the salt stress-induced change of apoplastic redox status may affect the formation of homo- and hetero-dimers of LRX3/4/5 and also affect the affinity of LRX3/4/5 proteins with RALFs, which finally transduce salt stress signals to the intracellular signaling pathways.

## 5. The Impact of Apoplastic pH on Salt Tolerance

In the early 1970s, the acid growth theory was proposed, which states that acidification of the apoplast promotes cell elongation, whereas alkaline state in the apoplast prevents cell growth [109]. The reduction of apoplastic pH (_apo_pH) activates several cell wall proteins, including expansins and other remodeling enzymes, resulting in the loosening of cell wall [110]. _apo_pH in linear growing cells is regulated by plasma membrane-localized H^+^-ATPases (AHAs) [111]. RALFs are a class of peptides that cause the alkalinization of the apoplast by regulating H^+^-ATPases via *Catharanthus roseus* RLK1-like kinases (*Cr*RLK1Ls). FER is one of the *Cr*RLK1L family proteins that consist of two carbohydrate-binding malectin-like domains, a transmembrane domain, and an intracellular serine/threonine-kinase domain [112,113]. FER inhibits the proton transport activity of AHA2 likely via direct phosphorylation [114]. It is known that salinity triggers the transient alkalization in the apoplast and inhibits plant growth [115], and our study showed that salt stress can induce the formation of mature RALFs [10]. These data suggest that salt stress-induced alkalinization of the apoplast is probably mediated by RALFs-FER-AHA2 module and the acidification of the extracellular environment is important for salt tolerance. Two halophyte species, *Atriplex lentiformis* and *Chenopodium quinoa*, which have a capacity to tolerate a high concentration of sodium ion, display a high H^+^-ATPase activity under salt stress, which contributes to a low _apo_pH and fast Na^+^ efflux [116]. *SOS1*, encoding a plasma membrane membrane-localized Na^+^/H^+^ antiporter, is required for the extrusion of excessive Na^+^ from the cytosol [117]. The Na^+^/H^+^ exchange activity of SOS1 is absent under normal growth conditions. Upon salt stress, however, Na^+^-induces induced formation of an ATP-dependent pH gradient can enhance the Na^+^/H^+^ transport activity of SOS1 [118]. Altogether, low _apo_pH facilitates plant growth under salt stress, but the direct effects of low _apo_pH on cell wall networks need more detailed studies.

## 6. Cell Wall Integrity Sensing and Signal Transduction under High Salinity

Unlike the traditional activation of plant receptor-like kinases by the corresponding ligands, the sensing of CWI is not limited by ligand-receptor pattern, e.g., recognition of wall fragments released from the damaged cell walls by receptor-like kinases, and is probably also achieved via the recognition of the cell wall modifications and the alteration of redox and _apo_pH status. Currently, a series of plasma membrane-localized receptor-like kinases and cell wall glycoproteins have been identified that are involved in the sensing and maintenance of CWI. As a universal signal molecule, Ca^2+^ is also involved in the transduction of CWI signaling signals in plants.

The cell wall appears to be the largest source of Ca^2+^ in plant cell [119]. Under normal conditions, Ca^2+^ is used to stabilize pectins via the dimerization of HG chains [120]. AGPs have been shown to bind abundant Ca^2+^ [121]. Under salt stress, the excessive accumulation of Na^+^ in the apoplast disrupts ion homeostasis, leading to rapid sodium-specific calcium waves occurred in roots [122]. The imported calcium ions directly bind the EF hands of RbohD/F and improve their catalytic activity [123,124]. Ca^2+^ is also an initial signal to activate the SOS signaling pathway, which promotes the extrusion of Na^+^ from the cytosol [125,126].

In addition to high salinity, other abiotic stresses, such as drought, cold, and osmotic stress, can also induce the cytosolic Ca^2+^ influx within a few seconds to minutes. Although the induction of Ca^2+^ signaling is a common event for these different abiotic stresses, studies have shown that the different stresses-triggered Ca^2+^ influx is mediated by different components. Reduced hyperosmolality-induced [Ca^2+^]_i_ increase 1 (OSCA1) is specifically required for the osmotic stress-triggered uptake of Ca^2+^ [127], and hydrogen-peroxide-induced Ca^2+^ increases 1 (HPCA1) is required for H_2_O_2_-, but not for salt- and osmotic stress-, induced influx of Ca^2+^ [128]. Glycosyl inositol phosphorylceramide (GIPC) sphingolipids, which are glycosylated via glucuronosyltransferase MOCA1, was discovered as a sensor of extracellular salt by directly binding to sodium ions [129]. The *moca1* mutant lacking functional GIPCs is defective in the activation of Ca^2+^ waves when being exposed to high concentration of Na^+^, K^+^, or Li^+^ ion. GIPCs can bind Na^+^ to gate Ca^2+^ influx channels and trigger the activation of SOS signaling pathway. However, which Ca^2+^ channels are activated by GIPCs and the mechanism underlying the activation need further study (Figure 1).

FER is considered as a CWI sensor and required for the activation of Ca^2+^ influx and maintenance of CWI under salt stress [9]. Mutation of *FER* reduces salt-induced Ca^2+^ influx in the root epidermis and increases sensitivity to high salinity. FER contains two malectin domains that have been experimentally demonstrated to directly bind with de-methyl-esterified HG in vitro and in vivo [9,130], suggesting that FER probably senses the cell wall changes directly via its extracellular domain and then transduces the cell wall signals to cellular interior via its cytoplasmic kinase domain. However, how the modification of pectin affects the activity of FER is still elusive. Our recent study showed that LRX3/4/5, RALFs, and FER function as a module to regulate salt stress response, which implies that FER-mediated perception of CWI probably needs the aid of LRX3/4/5-RALFs regulatory module [10]. Salt stress may dissociate the LRX3/4/5-RALFs complex via the salt stress-induced redox and pH changes in the apoplast, and the released RALFs bind to LLG1-FER complex and thereby allow the transduction of cell wall signals. The mechanism behind the dissociation of LRX3/4/5 and RALFs under salt stress needs to be further investigated.

THESEUS1 (THE1) is a *Cr*RLK1L family protein that was first identified in a screening for the suppressors of *prc1-1* [131]. The null mutation of *the1* partially suppresses the stunted growth and lignin deposition of the *prc1-1* mutant, despite the reduced cellulose content in the *prc1-1* is not restored [131]. HERKULES1 (HERK1) is another *Cr*RLK1L protein that is phylogenetically closely related to FER and THE1. Double mutant *herk1 the1-4* displays similar phenotypes as *fer-4* in terms of growth and salt stress response [52]. A recent study indicates that THE1 acts as the receptor of RALF34 to fine-tune lateral root initiation [132]. These results suggest that FER, THE, and HERK1 may work together to replay RALFs-mediated cell wall signals, but the biochemical associations among these three *Cr*RLK1L proteins are still largely unknown.

Male discoverer 1-interacting receptor like kinase 2 (MIK2) is a leucine-rich repeat receptor-like kinase (LRR-RLK) that was identified by a genome-wide association study (GWAS) based on the natural variations in response to salinity stress [133]. MIK2 controls root growth direction under salt stress in a THE1-dependent manner [134]. The salt-hypersensitive phenotype of *mik2* mutant can be suppressed by *the1-1*, a null mutation of *THE1* [134]. Recently, the serine rich endogenous peptide (SCOOP) phytocytokines were identified as the ligands of MIK2 to trigger immune responses [135], but whether the SCOOP peptides participate in MIK2-mediated regulation of salt tolerance is still unknown. FEI1 and FEI2 are two LRR-RLKs that are associated with cellulose synthesis and anisotropic cell expansion and are involved in CWI sensing [136]. Double mutant *fei1 fei2* displays root swelling and reduced cellulose biosynthesis under high sucrose or high salt conditions [137]. Genetic analysis indicated that FEI2 functions downstream of THE1 in mediating CWI perception [138]. Mid1-complementing activity 1 (MCA1) is a plasma membrane–localized stretch-activated Ca^2+^ channel and functions downstream of THE1 in *Arabidopsis* [95,139]. Like *the1-1* mutant, *mca1* seedlings exhibit reduced deposition of lignin and decreased jasmonic acid and salicylic acid biosynthesis in response to isoxaben-induced CWD [138].

Wall-associated kinases (WAKs) are a family of receptor-like Ser/Thr kinases whose extracellular domains are cross-linked with pectin fraction in a high affinity [140,141]. The EGF-like domain of WAK1/2 preferentially binds to de-methyl-esterified HG over methyl-esterified HG, and WAK1 also exhibits a high affinity with oligogalacturonides (OGs) in vitro [140,142]. The binding of WAKs to pectin and OGs occurs only in the presence of Ca^2+^ [140]. GRP-3, a glycine-rich cell wall protein, also acts as a potential switch for the kinase activity of WAK1 and negatively regulates the defense responses elicited by OGs [143]. A dominant allele of *wak2* mutant exhibits constitutive activation of stress responses, including increased ROS accumulation and cell wall biogenesis [142,144]. Under long-term salt stress, tomato *WAK1* mutant *slwak1* exhibits disrupted osmotic homeostasis and elevated sucrose content in roots, which in turn negatively affects fruit yield [145]. Similarly, *Ds* transposon insertion mutant of *HvWAK1* in barley displays decreased salt tolerance [146]. Although the WAKs have been shown to participate in the salt stress response, the existing experimental evidences to elaborate the roles of WAKs in sensing the CWI under salinity are still lacking. Recently, Gigli-Bisceglia et al. indicated that salinity stress-induced de-methyl-esterification of pectin activates stress signaling pathways, which may provide a direction to study the roles of WAKs in salt stress response [52] (Figure 1).

The CWD caused by salinity stress, isoxaben, an inhibitor of cellulose biosynthesis, or driselase, a cell wall-degrading enzyme, can increase the protein levels of hormone-like peptides PROPEP1/3, the precursors of plant elicitor peptide 1/3 (Pep1/3) [138,147]. The *Pep3* knockdown plants and the null mutant of *Pep1 receptor 1* (*PEPR1*) both exhibit salt-hypersensitivity [148]. These results suggest that the activation of PEPR1 by PROPEP3 positively regulates salt tolerance in *Arabidopsis*. Currently, the majority of studies on Peps-PEPRs complexes focus on their roles in plant immunity, and in future the roles of the Peps-PEPRs complexes-mediated signaling in abiotic stress responses need more investigations.

HPCA1 is a LRR-RLK required for the sense of extracellular H_2_O_2_ [128]. The two pairs of cysteine residues in the extracellular domain of HPCA1 are covalently modified by extracellular H_2_O_2_, which leads to the activation of HPCA1 and elevation of Ca^2+^ influx. In *hpca1* mutant seedlings, the extracellular H_2_O_2_-induced Ca^2+^ influx, the activation of ABA signaling, and the phosphorylation of MPK3/6 are all inhibited [128]. It was shown that HPCA1 is not required for the salt stress-induced influx of Ca^2+^, but considering that high salinity can affect the redox status in the apoplast, so whether HPCA1 is also required for the sense of salt stress-induced redox changes worth further investigations.

## 7. Salt Stress-Triggered Intracellular Signaling Pathway Regulated by Cell Wall Sensors

Although several plasma membrane-localized cell wall integrity sensors have been identified that perceive cell wall changes, the intracellular signaling pathways that relay cell wall signals are still largely unknown. The phosphorylation of MPK6 is a marker of the environmental stimuli, and the transient activation of MPK6 under abiotic stress conditions, including high salinity and cold, has been reported [149]. As a major signaling transducer, the activity of MPK6 is regulated by multiple CWI sensors, such as FER, THE1, HERK1, and HPCA1 [52,128]. In future, the regulatory mechanisms of these CWI sensors on the activity of MPK6 need to be addressed.

After perception of CWD by cell wall sensors, plants can integrate and balance multiple hormone signals to improve salt tolerance. ABA and JA are the major hormones involved in the response to diverse environmental stresses. In the *lrx345* and *fer-4* mutants, the ABA and JA contents are constitutively increased and the salt-hypersensitivity of these two mutants is largely caused by the disrupted homeostasis of phytohormones [150]. Phosphatase ABA insensitive 2 (ABI2) is a negative regulator of ABA signaling pathway, and FER activates the guanine nucleotide exchange factor (GEF) 1/4/10/Rho of plant 11 (ROP11) pathway to positively regulate the activity of ABI2 phosphatase, and thereby modulating ABA signaling pathway [151,152]. MYC2, a master transcription factor in JA signaling pathway, is also regulated by FER. FER positively regulates immunity by inhibiting JA signaling via the phosphorylation-mediated destabilization of MYC2 [153]. It has also been shown that MYC2 negatively regulates salt tolerance via the inhibition of proline biosynthesis [154]. In brief, these results suggest that FER controls the environmental stress responses via the modulation of the homeostasis of phytohormones (Figure 1).

## 8. Cell Wall Repair under High Salinity

Upon exposure to salt stress, the cortical microtubules in the hypocotyl of seedling are rapidly depolymerized, the process of which usually occurs within 2 h of salt application. However, at the growth recovery stage (after salt treatment for ~8 h), the cortical microtubules are reassembled into stable cortical arrays [16]. Evidences have shown that the rapid depolymerization of the cortical microtubules network is important for salt tolerance. For instance, stabilization of microtubules with paclitaxel leads to increased salt-hypersensitivity, whereas constitutive disruption of microtubules with oryzalin or propyzamide improves salt tolerance [155].

The depolymerization of cortical microtubules requires the alteration of the activities of the atypical microtubule-associated protein kinase propyzamide hypersensitive 1 (PHS1) and microtubule-associated protein SPIRAL1 (SPR1). Under normal growth conditions, the kinase activity of PHS1 is inhibited by its own phosphatase domain, while salt or osmotic stress blocks this inhibition and then enhances the phosphorylation and depolymerization of α-tubulin [156]. SPR1 binds to the microtubules and antagonizes stress-induced cortical microtubule depolymerization. Under salt stress, SPR1 is rapidly degraded by the 26S proteasome and the inhibition of microtubule depolymerization is relieved [157]. Histone H2B monoubiquitination (H2Bub1) participates in the regulation of the expression of *protein tyrosine phosphatase 1* (*PTP1*) and *MAP kinase phosphatase* (*MKP*) genes, which in turn modulate the phosphorylation and dephosphorylation of microtubule-binding proteins via a PTP1/MKP-MPK3/6 signal mode, and finally promotes the rapid microtubule depolymerization under salt stress [158].

CSCs synthesize cellulose via the binding with cortical microtubules, and the polymerization status of cortical microtubules determines the movement of CSCs at the cell surface. CSCs are assembled in the Golgi apparatus and translocated to the plasma membrane via vesicle trafficking. Salt-induced depolymerization of microtubules is accompanied by the internalization of CSCs into small CesA compartments/microtubule-associated CesA compartments (smaCCs/MASCs) [15]. At the growth recovery stage after salt treatment, cortical microtubule is reassembled and CSCs is relocated to the plasma membrane to synthesize cellulose. Increasing evidences have shown that the efficiency of plants to reassembly cortical microtubule and cellulose during the growth recovery stage is critical for salt tolerance. CC1 and its paralog CC2 were identified as companions of CSCs and are required for the reassembly of cortical microtubule and subsequently cellulose biosynthesis during the growth recovery stage [16]. In *cc1 cc2* double mutants, CSCs dissociate from the microtubules after salt treatment, but a stress-tolerant microtubule complex cannot be reproduced, resulting in the abolishment of the localization of CSCs at the plasma membrane and decreased cellulose synthesis. Microtubules-associated proteins 65-1 (MAP65-1) is a plant microtubule-bundling protein, which participates in the polymerization and bundling of cortical microtubules [159]. Phosphatidic acid (PA), a product of phospholipase D (PLD), binds to MAP65-1 and increases its activity to enhance microtubule polymerization and bundling [160]. The *pldα1* mutant exhibits a defect in microtubule organization under salt stress and increased salt-hypersensitivity. Moreover, 16:0–18:2 PA can activate MPK6 via directly binding to MPK6 and the salt-induced transient activation of MPK6 is abolished in the pldα1 mutant [149].

Brassinosteroid insensitive 2 (BIN2), a master negative factor in brassinosteroid signal pathway, regulates the balance between salt stress response and growth recovery [161]. BIN2 is required for the negative regulation of cellulose biosynthesis. BIN2 phosphorylates CESA1 to inhibit the activity of CSCs [162]. By exploring turboID-mediated proximity labeling technology, Kim et al. found that BIN2 interacts with FER, but the biological significance of this interaction has not yet been resolved [163]. It is possible that FER regulates the activity of BIN2 via phosphorylation, and then modulates CesAs activity and cellulose biosynthesis under salt stress.

## 9. Transcriptional Regulation of Cell Wall-Associated Genes under Salt Stress

Under salinity stress, plant cells sense salt signals via receptors or sensors and then transmit the signals to the downstream regulatory networks to trigger the transcription of salt stress-responsive genes, which in turn promote the adjustment of the physiological, biochemical, and metabolic properties of plant cells to adapt to high salinity.

The transcriptional regulation of genes largely depends on the activity of the corresponding transcription factors. Some transcription factors have been identified that are required for the regulation of cell wall-associated genes in response to salt stress. For example, salt stress induces the accumulation of β-1,4-galactan in root cell walls through the up-regulation the of *galactan synthase 1* (*GALS1*) gene. Based on a genetic screening, two transcription factors basic pentacysteine 1 (BPC1) and BPC2 were identified that directly bind to the promoter of the *GALS1* gene and repress its expression [28]. The expression of *BPC1* and *BPC2* genes is significantly reduced under salt stress. The *bpc1 bpc2* double mutant, in which the accumulation of β-1,4-galactan is elevated under salt stress compared with the wild type, exhibits increased salt tolerance [28]. *Oryza sativa MULTIPASS* (*OsMPS*) encodes an R2R3-type MYB transcription factor in rice. Expression profiling revealed that, upon ABA or salt stress treatment, the expression of expansins, such as *OsEXPA4*, *OsEXPA8*, *OsEXPB2*, *OsEXPB3*, and *OsEXPB6*, and the expression of cell wall biosynthesis genes, such as endoglucanase genes *OsGLU5* and *OsGLU14*, are negatively regulated by OsMPS [164]. *XTH19* and *XTH23*, belonging to xyloglucan endotransglucosylase/hydrolase group II, are up-regulated by salt stress and BR [43]. In the *xth23* single or *xth19 xth23* double mutant, lateral root growth is disrupted under salt stress, whereas overexpression of *XTH19* or *XTH23* enhances salt tolerance and increases lateral root initiation [43]. BRI1-EMS-SUPPRESSOR 1 (BES1) is a transcription factor that is involved in BR signaling pathway. BES1 directly binds the promoter of *XTH19* and *XTH23* and positively regulates their expression under salt stress [43] (Table 1).

Gene expression is also influenced by epigenetic regulation, such as histone modification and DNA methylation. Salt stress triggers the histone H3K9/K14 acetylation of some abiotic stress-responsive genes to crease their transcript levels [165]. *General control nonderepressible 5* (*GCN5*), encoding a histone acetyltransferase, is induced by salt stress and acts as a maintainer of CWI. GCN5 mediates the acetylation of H3K9 and H3K14 in the promoters of *CTL1*, *PGX3* (*polygalacturonase involved in expansion-3*), and *MYB54* under salt stress, and thus fine-tunes their gene expression [32]. Constitutive expression of *CTL1* partially restores the salt-hypersensitivity and CWD of the *gcn5* mutant [32]. Similarly, the H3K9 acetylation level in the genome of maize is also elevated after salt treatment, and the increased acetylation level enhances the expression of *ZmGCN5*, which in turn promotes the expression of *ZmEXPB2* and *ZmXET1* genes [166].

## 10. Conclusions and Future Perspective

Cell wall is not just a mechanical support for plant cells, but is also the frontline to sense and transduce environmental stress signals. High salinity, as one of the globally distributed abiotic stresses, can disrupt the CWI, and the severity of the salt-triggered CWD largely depends on the concentration of the surrounding sodium ion combined with other environmental conditions, such as light intensity and water availability. Study of the mechanisms underlying the sensing and maintenance of CWI under salt stress not only strengthens our understanding of salt stress responses in plants but also provides new strategies for the cultivation of crops with improved salt tolerance. Regarding the associations between CWI and salt tolerance, there are still many questions remain to be addressed, and the most important ones could be that how the excessive accumulation of Na^+^ in the apoplast affects the CWI, and how the salt-induced cell wall changes are sensed by the cell wall sensors. Moreover, the Ca^2+^ channels that are required for the relay of salt-triggered cell wall stress signals need to be identified and the cell wall repair mechanisms under stress conditions need to be further investigated. With the development of gene editing technologies and improved transformation efficiency, editing of CWI-related genes in crops to generate salt-tolerant varieties can be applied in future.

## Figures and Tables

**Table 1:** List of the cell wall biosynthesis-related genes that are involved in salt stress response.

Name	Gene ID	Annotation	Function	Reference(s)
*AtCesA1/RSW1*	At4g32410	Cellulose synthase catalytic subunit	Cellulose synthesis in the primary cell wall	[22]
*AtCesA8/IRX1*	At4g18780		Cellulose synthesis in the secondary cell wall	[25]
*AtCC1*	At1g45688	Cellulose synthase companion protein	Cortical microtubules assembly and cellulose biosynthesis under salt stress	[16]
*AtCC2*	At5g42860			
*AtCTL/POM1*	At1g05850	Chitinase-like protein 1	Involved in the assembly of glucan chains	[24,26]
*AtCSI1/POM2*	At2g22125	Cellulose synthase-interactive protein 1	Companion of CesAs; required for cell elongation in root	[21]
*AtCCoAOMT1*	At4g34050	Caffeoyl-CoA 3-*O*-methyltransferase	Involved in lignin synthesis	[17]
*AtKOR/RSW2*	At5g49720	Endo-β-1,4-glucanase	Integral component of CSC; required for cell elongation in root	[23]
*AtHSR8/MUR4*	At1g30620	Golgi-localized UDP-D-xylose 4-epimerase	Arabinose biosynthesis; related to the modification of polysaccharides and glycoproteins	[27]
*AtGALS1*	At2g33570	β-1,4-galactan synthase	Transfer of galactose from UDP-α-d-Gal or arabinopyranose from UDP-β-l-Ara*p* to growing β-1,4-galactan chains	[28,29]
*AtXTH30*	At1g32170	Xyloglucan endotrans glucosylase-hydrolase	Cleave or rejoin the xyloglucan; *xth30* mutation decreases crystalline cellulose content and affects the depolymerization of microtubules under salt stress	[30]
*AtPMEI13*	At5g62360	Pectin methyl-esterase inhibitor 13	Inhibits the activity of PMEs	[31]
*AtBPC1*	At2g01930	BPC-type transcription factor	Regulation of the expression of *AtGALS1*	[28]
*AtBPC2*	At1g14685			
*AtGCN5*	At3g54610	Histone acetyltransferase	Epigenetic regulation of cell wall-related genes	[32]
*OsTSD2*	Os02g51860	Pectin methyltransferase	Regulation of pectin metabolism	[33]
*OsBURP16*	Os10g26940	β subunit precursor of polygalacturonase 1	Involved in cell wall pectin degradation	[34]

## Data Availability

Not applied in this study.
